# Solicitation matters: Cultural differences in solicited and unsolicited support provision

**DOI:** 10.3389/fpsyg.2022.953260

**Published:** 2022-10-19

**Authors:** Hirofumi Hashimoto, Takuma Ohashi, Susumu Yamaguchi

**Affiliations:** ^1^Graduate School of Literature and Human Sciences, Osaka Metropolitan University, Osaka, Japan; ^2^Graduate School of Humanities and Sociology, The University of Tokyo, Tokyo, Japan

**Keywords:** social support, cultural differences, cultural psychology, unsolicited support, solicited support

## Abstract

Two studies aimed to examine cultural differences in social support provision, with or without solicitation, in Japan and the United States (US). In Study 1, we replicated a previous study with Japanese university students. We found that the Japanese participants did not provide social support when it was not solicited, as compared with when it was solicited. Furthermore, in Study 2, participants were asked to respond to a questionnaire regarding a hypothetical stressful situation experienced by a close other and to indicate their willingness to provide support. We confirmed our hypothesis that Japanese participants hesitate to provide unsolicited support to close others (such as family members or close friends), even when they recognize that the close others are in need, whereas the American participants do not hesitate to provide such support. Contrastingly, regarding solicited support, the Japanese and Americans were equally ready to provide support, as hypothesized. The cultural difference in social support resides in the provision of unsolicited support. These results suggest that differences in culturally appropriate responses to needy people are responsible for the difference in the provision of unsolicited vs. solicited social support.

## Introduction

In daily life, we occasionally encounter situations where someone is in need of personal help. If we notice that someone else is experiencing a difficulty, we may consider helping that person coping with their problem. The term “social support” has long been attracting attention in social psychology to refer to this kind of everyday personal help. Social support is defined as a form of support that leads the individual to believe that they are cared for, loved, esteemed, and a member of a network of mutual obligations (Cobb, 1976). A central and traditional topic in social psychology on social support is the study of the stress-buffering effect that social support provides (e.g., Cohen and Wills, 1985). In the 1990s, cultural psychologists began to argue that there could be cultural differences in social support. Specifically, it has been shown that East Asians and/or Asian Americans are less likely than European Americans to seek support from others in stressful situations (e.g., Taylor et al., 2004).

If social support is associated with positive outcomes (i.e., stress-buffering effect) as argued by Cohen and Wills (1985), a question arises on what hinders East Asians in need from seeking help from others. One possibility is that the sense of “relational concern” (Taylor et al., 2004; Kim et al., 2006) is more pronounced among East Asians, that is, they tend to be reluctant to seek social support to cope with their difficulties and stresses because they are concerned that their accruing potentially negative reputation could burden their group harmony (Kim et al., 2006). This standard interpretation by cultural psychologists is well-taken in the field. In the present study, we extend the implications of relational concern to support providers’ psychological mechanisms. As a matter of course, social support is an act of mutuality between the support-provider and the support-recipient. However, previous studies on social support, focusing on relational concern, has been slanted toward the support-recipient’s perspective.

Chen et al. (2012) exceptionally focused on support providers’ motivations. Based on their findings, they suggested that support provision in Western European countries is generally intended to enhance the support recipient’s self-esteem, whereas, in East Asian countries, support provision is intended to achieve closeness with the support recipient. They further suggested that these cultural differences in social support provision reflect differences of self-construals (Markus and Kitayama, 1991). Those with interdependent self-construals, in which the self is formed by establishing harmonious relationships, may consider the intimacy of those relationships when offering social support to others. It is conceivable that those who hold this interdependent self-construal, in which the self is formed by establishing harmonious relationships, would consider the intimacy of those relationships when offering social support to others.

Considered together, Taylor et al.’s (2004) and Chen et al.’s (2012) findings suggest that East Asians take interpersonal relations into consideration, such that their support does not disrupt interpersonal harmony with the support recipient. Taylor et al.’s (2004) study reports that people in East Asian cultures hesitate to seek social support because they are concerned about bringing their personal problems to other’s attention. Asian support providers are motivated to provide their support to achieve closeness with the person in need (Chen et al., 2012). Then, how does relational concern lead East Asians to behave in relation to the interactions between people in need and potential support providers? To answer this question, a “cultural game player” perspective would be useful (Hashimoto et al., 2011; Hashimoto, 2019). According to this perspective, culturally unique behaviors represent adaptive strategies rather than simple expressions of personal psychological mechanisms. These behaviors are often tailored to enable players to succeed in socially adaptive tasks and thereby obtain valuable resources from others. Regarding the provision of social support in a culture with relational concern (Taylor et al., 2004; Kim et al., 2006), we assumed that in East Asian countries, social support would be less likely to be sought and consequently, more likely to be provided hesitantly, unless it was solicited explicitly. This is because potential support providers would anticipate the possibility that people in need are not necessarily eager to be helped owing to their relational concern and thus, offering support may have an adverse effect on their psychological well-being. It is possible that for those people social support is embarrassing and thus, they may respond to the support provider negatively. This way, the psychological mechanism of relational concern matters not only for support recipients, but also for potential support providers.

In summary, we hypothesized that potential support providers in East Asian countries would be hesitant and less likely to provide social support when it is not solicited, because they expect an unpleasant response from the support recipients in such situation. This reasoning, based on the cultural game player perspective, differs from Chen et al. (2012), who did not take into consideration an inhibitive factor in social support provision caused by relational concern. In other words, solicitation is assumed to play an important role in the provision of social support in East Asia, where people hesitate to provide support unless explicitly solicited. To examine our hypothesis, we adopted a method used by Chen et al. (2012), which assumes a certain level of support provision, and examined the relation between solicitation and provision of support. Additionally, Chen et al. (2012) listed motivation to closeness and to restore the self-esteem of the distressed person as major motivations for providing social support. We expected that the importance of interpersonal relationships would motivate one to restore the psychological well-being of a person in distress. A possible operation of this motivation was examined in Study 1 by adding additional question items. Furthermore, in Study 2, we examined the differences between Japan and the U.S. in the effect of solicitation using the hypothetical scenario method.

## Study 1: Solicitation and provision of support in Japan

### Methods

#### Participants

One hundred and eighty-three Japanese undergraduates (123 females and 60 males, mean age = 19.26 years) participated in the study.^1^

#### Procedures

To examine our hypothesis, we modified the questionnaire used in Chen et al. (2012). The questionnaire asked participants to retrospect on the last 3 months, and to recall and describe a highly stressful recent event experienced by a close other (a friend or family member). Then participants were asked, “Did you actually support someone close to you who was experiencing a highly stressful event?” After they described the event, they were also asked to indicate the extent to which three types of motivation behind their social support provision were applicable. Two of the three types of motivation scales were used by Chen et al. (2012): *motivation for closeness,* measured with three items (such as “I wanted my close other to feel close to me”) and *motivation for self-esteem,* measured with three items (such as “I wanted my close other to have high self-esteem”). These two types of motivation are consistent with the discussion of cultural self-construals (e.g., Markus and Kitayama, 1991) and worthy of consideration. However, it should be noted that the mean values for Japanese participants on the scale measuring the two types of motivation are below the midpoint in Chen et al. (2012). This result suggests that these two types of motivation may not be dominant motives underlying the provision of support in Japanese participants. In Study 1, therefore, we added three new questions. First, in terms of motivation for social support, we created and added the *motivation for restoration of well-being* (such as “I wanted my close other to live a happy life like they did before”). Here, we expected the observed tendency for Japanese participants to score lower on the *motivation for closeness* and *motivation for self-esteem* to be due to the limited focus, and assumed by focusing on restoring a more general well-being the motivation for support could be accessed. Second, we added a new item related to hesitation in providing support (specifically, “I thought it was better not to provide support unless my close other explicitly asked me for help”) to the questionnaire so that we could examine inhibitors as well as facilitators for support provision (*hesitation*). Here, we expected that Japanese participants would tend to be hesitant about providing social support unless it was solicited by the person in need. Third, we also included a questionnaire item asking whether they were explicitly solicited to provide social-support by the close other in the event they recalled. Based on our hypothesis, we expected that participants would tend to not provide support unless it was solicited.

As shown in the Results section, we used the chi-square test to assess the relationship between the presence or absence of solicitation and the social support provision to examine our hypothesis. Furthermore, as an exploratory analysis, we also divided the groups after we checked the participants’ response to whether they provided support or not and whether they were asked to support or not, and compared the scores regarding motivation scales between these groups by using t-tests and ANOVAs. In particular, we focus on the scores of items related to hesitation.

### Results and discussion

**Figure 1:**
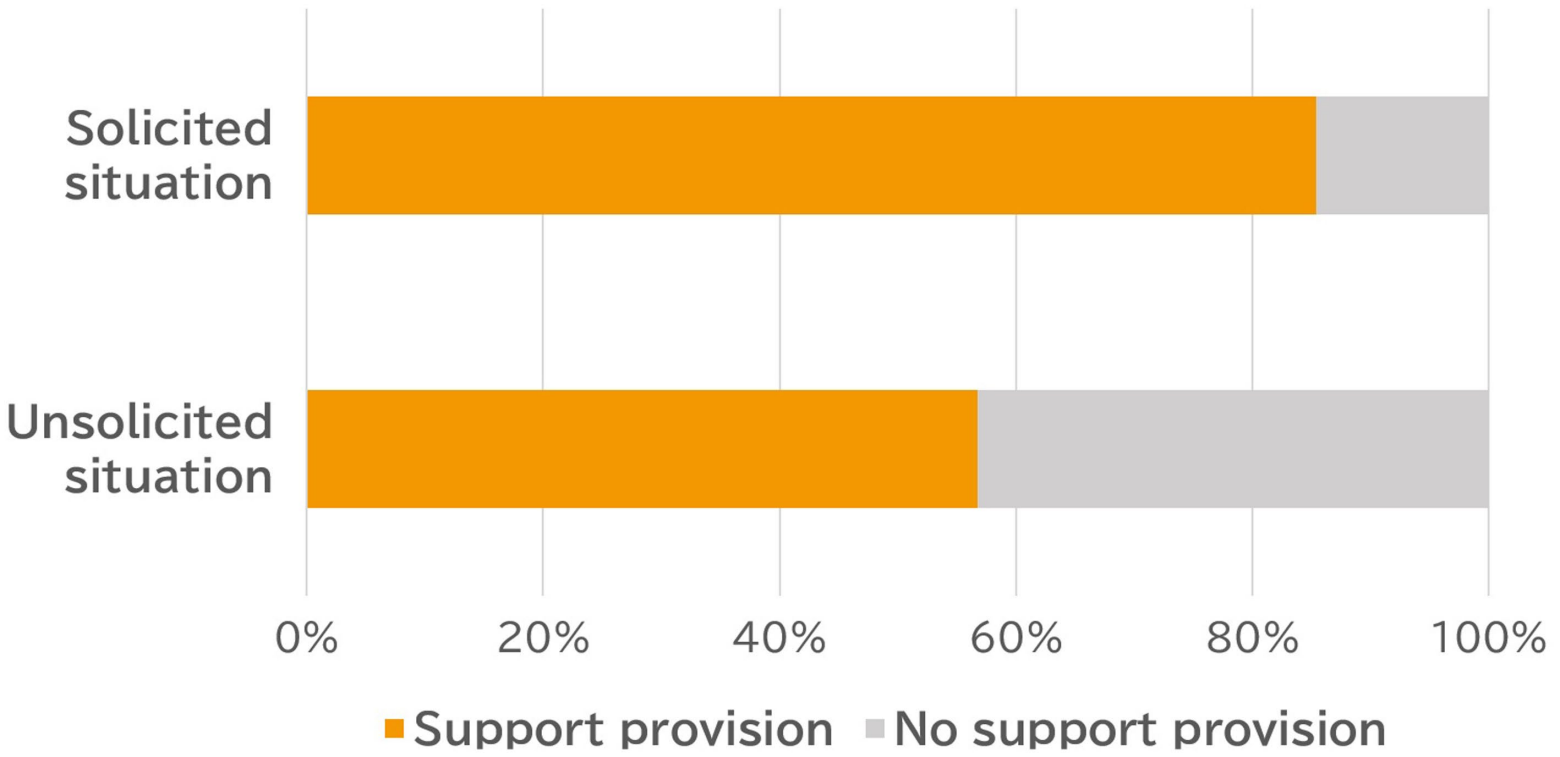
Provision rate of solicited and unsolicited social support among Japanese participants.

The relation between solicitation and provision of support is shown in Figure 1. As can be seen, the Japanese participants did not provide support when it was unsolicited as compared with when it was solicited (Chi-square test: *χ*^2^(1) = 11.27, *p* < 0.001), indicating that the presence or absence of solicitation for support has a significant effect on Japanese people’s provision of support, even when they are aware that their close other is distressed.^2^ This result provides a clear support for our hypothesis. In addition, our exploratory analysis demonstrated that those who did not provide support were more hesitant (*M* = 4.18) as compared with those who provided support (*M* = 3.27, *t*-test: *t* (173) = 3.52, *p* < 0.001).^3^ Furthermore, among those who reported that they were not explicitly asked to provide social support by their close others, there was a significant difference in *hesitation* between support providers (*M* = 3.29) and non-providers (*M* = 4.21; *t* (125) = 3.24, *p* = 0.002), which supports our hypothesis as well.^4^

The findings in Study 1 provide a coherent picture of social support provision in Japan. Japanese people are highly influenced by the presence or absence of support solicitation from the recipients when they make a decision to provide personal support to distressed people. However, in Study 1, participants were just asked to freely recall situations in which a close other was in a stressful situation. Thus, there remains a possibility that these situations differed among the participants. In Study 2, we controlled for such differences by using a scenario method. In addition, Study 2 examined if Japanese participants would attempt to take care of close other’s psychological well-being based on their nuanced social interactions, to the extent that they would hold provision of social support until the distressed close other explicitly solicited support. Previous studies (Taylor et al., 2004; Kim et al., 2008) have found that Japanese people hesitate to solicit support, a tendency that was also confirmed by Study 1. The participants in Study 1 (72.6%; 127/175) tended to report that they were not explicitly asked for social support by their close others. This result can be interpreted as an observation that solicitation of social support is uncommon among Japanese people, even among those who are close to each other. People in distress may hesitate to solicit support and consider it embarrassing to do so; however, one cannot restore psychological well-being when support is not solicited. If Japanese people take such psychological interactions into consideration as the cultural game player perspective suggests, the findings in Study 1 would not be replicated in the U.S. Study 2 examined cultural differences in response to the presence/absence of solicitation for support by controlling the situation using a scenario method. We hypothesized that the presence/absence of solicitation would affect Japanese participants’ provision of support but not Americans’.

## Study 2: Examination of cross-cultural differences

### Methods

#### Participants

One hundred and eighteen Japanese undergraduates (26 females and 92 males, mean age = 19.92 years) and 52 European–American undergraduates (36 females, 15 males and one unspecified, mean age 20.73 years) participated in the study (See footnote 1).

#### Procedures

Participants were given a questionnaire and asked to answer at their own pace. The questionnaire began with the following statement: *Most people encounter stressful events (such as relationship problems, financial difficulties, conflicts with family members, illness, job stress, school-related concerns) on a fairly regular basis. In the following questions, we will ask about such stressful events in daily life.* Participants were then asked to imagine the following situation: *Recently, your close other experienced a very stressful event.* After reading this statement, participants were asked the following questions and then asked to respond with one of four responses: “*If your close other experienced a very stressful event, would you support him/her? Please do not answer in terms of whether you ‘should’ support, or whether you ‘would like to’ support, but think and answer ‘how likely’ you would actually support him/her in this situation based on your daily experiences” (1 = I would support him/her in any case, 2 = I would support him/her in many cases, 3 = I would not support him/her in many cases, 4 = I would not support him/her in any case)*. Since we are not including any information about the solicitation here, we will refer to this situation as the *no-information condition* below.

After answering this question, participants were asked to indicate the extent to which the three motivation types were underlying their social support provision, as in Study 1. The questionnaire further asked participants whether they would provide support in situations where there was clear support solicitation from a close other and in situations where there was not. The specific question statements were the following: *“Recently, your close other experienced a very stressful event, so he or she explicitly asked you to give support (Solicited condition)”* and *“Recently, your close other experienced a very stressful event. However, he or she did not explicitly ask you to give support (Unsolicited condition).”* For each condition, participants were asked to choose one of the four responses listed above.

The primary focus of our analysis in Study 2 was on cultural differences. Therefore, we examined our hypotheses utilizing chi-square tests to examine cultural differences in the social support provision for each scenario. As in Study 1, we also assessed cultural differences in the scores regarding motivation scales with t-tests, although these are exploratory analyses.

### Results and discussion

**Figure 2:**
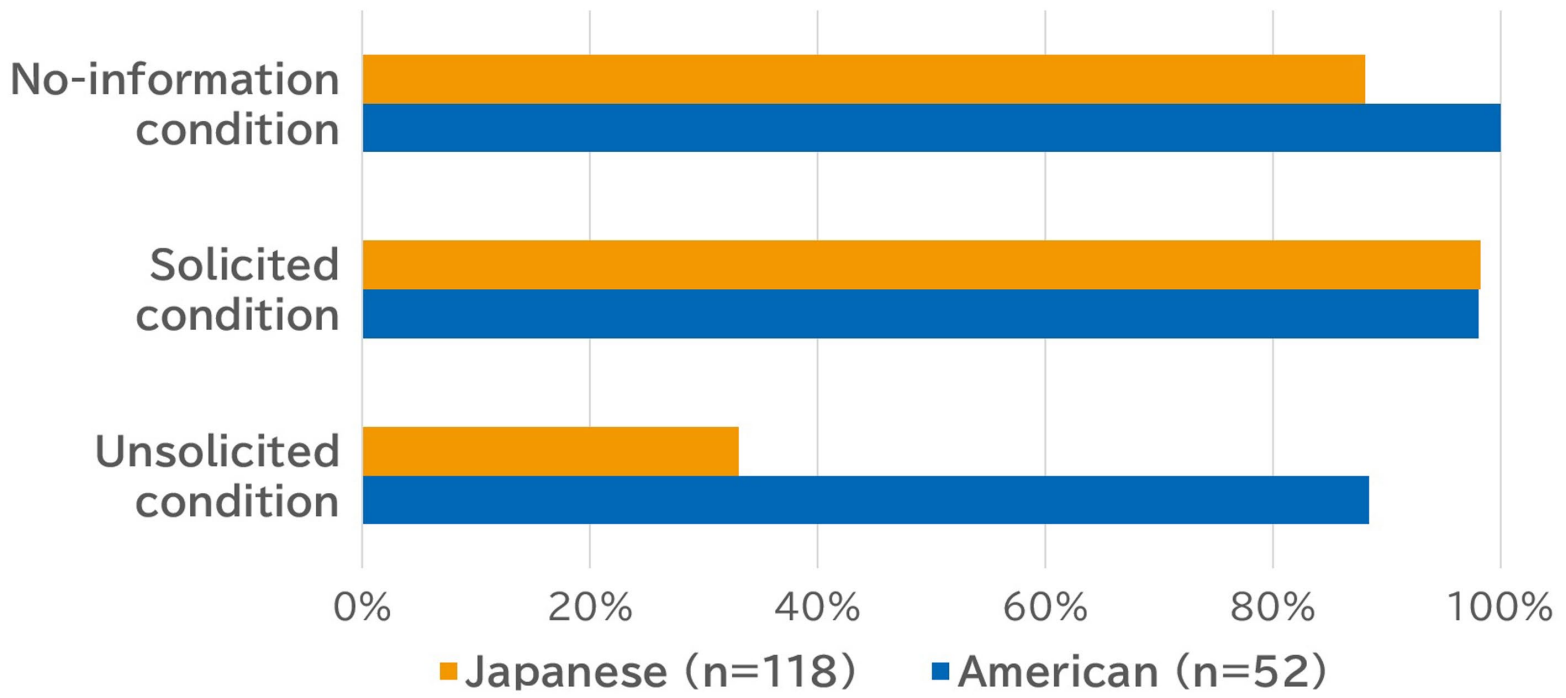
Provision rate of solicited and unsolicited social support among Japanese and American participants.

As shown in Figure 2, cultural differences in the rate of support provision were most salient in the unsolicited condition. That is, less than half of Japanese participants (33.05%) answered that they would offer support, whereas most American participants (88.5%) answered that they would offer support (Chi-square test: *χ*^2^(1) = 44.33, *p* < 0.001),^5^ providing a clear and strong support to our hypothesis. This difference disappeared in the solicited condition (*χ*^2^(1) = 0.01, n.s.), in which most participants from both cultures answered they would offer support. In the no-information condition, contrastingly, there was a smaller but significant difference, such that 88.1% of Japanese participants and all American participants returned a positive answer (*χ*^2^(1) = 6.72, *p* = 0.009).^6^ Some Japanese participants in this condition may well have assumed that the distressed person was not soliciting support.

The motivations underlying support provision were found to vary with culture. Table 1 shows the descriptive statistics of three measures of motivation and a measure of hesitation along with their reliability in each country. As can be seen, the restoration of psychological well-being is most stressed among Japanese participants (*F* (2, 232) = 103.38, *p* < 0.001), whereas it is not the case with American participants (*F* (2, 102) = 0.72, *p* = 0.490). This result suggests that Japanese people are more concerned with the distressed person’s psychological state than with simply providing support to remove the tangible burden of the distressed person. It is possible that Japanese people like to confirm that their support will not embarrass the recipient. In line with this reasoning, Japanese participants hesitated to provide support to a greater extent than their American counterparts (*M* = 4.57 vs. 2.58, *t* (167) = 7.59, *p* < 0.001).

**Table 1:** The reliability coefficients, means, and standard deviations of the four scales used to measure motivation for social support provision.

	Japanese		Americans	
	Mean	SD	Mean	SD
Motivation for closeness (*α*_JP_ = 0.84, *α*_US_ = 0.80)	3.93	1.50	6.44	0.76
Motivation for self-esteem (*α*_JP_ = 0.67, *α*_US_ = 0.87)	4.43	1.12	6.58	0.58
Motivation for restoration of well-being (*α*_JP_ = 0.86, *α*_US_ = 0.93)	5.76	0.83	6.48	0.90
Hesitation	4.57	1.57	2.58	1.59

## General discussion

The findings in Studies 1 and 2 provide a coherent picture of social support provision among Japanese people in comparison to American people. Japanese support providers are involved in a more nuanced psychological interaction with the potential recipient of social support. Apparently, Japanese people are eager to know in advance if their support will be welcomed by the potential recipient, as they tend to be motivated to restore the psychological well-being of the distressed other and they know there are people who do not wish to solicit support (to avoid disruption of interpersonal relationships). As a result, Japanese people hesitate to provide social support when it is not solicited by the distressed person, even when they are close to the person. This mental process is not found in the American culture, where people do not have rigid social norms (Gelfand et al., 2011) and are less worried about disrupting interpersonal relationships by receiving social support.

A psychological mechanism of hesitation in seeking and receiving social support has been shown in previous cultural psychological studies (e.g., Taylor et al., 2004; Kim et al., 2008; Mojaverian and Kim, 2013; Ishii et al., 2017). In the present study, we attempted to extend the psychological mechanism from the support recipient’s perspective to the provider’s perspective. Interestingly, the data from the present study demonstrate that providing and seeking social support among Japanese participants is more nuanced than it has been thought in previous studies. Distressed people are hesitant to seek support because they fear a potentially negative evaluation from the support provider. Similarly, the provider also expects a potentially negative evaluation from the support recipient and thus hesitates to provide support. This is consistent with the findings of Niiya et al. (2022), who also examined the provision of social support among Japanese people and found that it is highly dependent on the assessment of the needs of others. One could assume from these results that the “rejection avoidance” on both sides makes it difficult to support each other (Hashimoto and Yamagishi, 2013, 2016). Yamagishi and colleagues have argued that in long-term and “closed” relationships, where acceptance of needed resources from closely related providers is the primary condition for survival, being sensitive to these resource providers’ feelings and avoiding their rejection is an adaptive strategy (Yamagishi et al., 2008, 2012; Yamagishi and Hashimoto, 2016). In this sense, it is plausible that Japanese participants are less likely to seek and provide social support due to mutual avoidance of rejection. Obviously, this line of interpretation needs to be examined in a future study, by addressing important questions such as of “how” and “why” Japanese people provide their support according to the situation.

Several issues remain to be addressed in future research. First, because both Studies 1 and 2 measured only the verbal responses of the participants, future studies could examine people’s real social support in an experimental setting. Second, although the current study focused on the presence or absence of solicitation in discouraging social support provision, other potential factors need to be examined in more detail and reexamined using experimental procedures. Third, the cross-cultural comparison was limited to the difference between Japan and the US. Because it is known that Japanese youths have a strong tendency toward rejection avoidance (Hashimoto, 2021), it is possible that hesitancy to providing social support is a phenomenon unique to Japanese youths in East Asia. Future studies should examine if the present findings are unique to the Japanese youth. Despite these limitations, the present study contributes to our understanding of the cultural differences in social support. Japanese people’s provision of social support can be affected by factors absent in the West.

## Data availability statement

The raw data supporting the conclusions of this article will be made available by the authors, without undue reservation.

## Ethics statement

The studies involving human participants were reviewed and approved by The Research Ethics Committee of the Department of Social Psychology, Graduate School of Humanities and Sociology, The University of Tokyo. The patients/participants provided their written informed consent to participate in this study.

## Author contributions

HH and TO analyzed the data. HH and SY wrote the first draft of the manuscript. All authors developed the study concept and design and collected the data. All authors contributed to the article and approved the submitted version.

## Funding

This study was supported by Suntory Foundation Research Grants for Young Scholars and by a grant from Yoshida Hideo Memorial Foundation.

## Conflict of interest

The authors declare that the research was conducted in the absence of any commercial or financial relationships that could be construed as a potential conflict of interest.

## Publisher’s note

All claims expressed in this article are solely those of the authors and do not necessarily represent those of their affiliated organizations, or those of the publisher, the editors and the reviewers. Any product that may be evaluated in this article, or claim that may be made by its manufacturer, is not guaranteed or endorsed by the publisher.

## Footnotes

^1^We set a target sample size of 180 male and female Japanese participants in Study 1 based on the study of Chen et al. (2012), who collected data from 93 Japanese female college students. We initially determined the sample size in Study 2 in the same way as in Study 1. However, we could not collect sufficient data during the semester as planned originally. In Study 1, we did not include eight participants in part of the analysis because they did not respond to the question of whether they provided support. Additionally, the Japanese participants in Study 2, who participated in a different study, were asked to complete the present study’s questionnaire as an additional questionnaire. The findings of the other study have already been published.

^2^Considering the unbalanced sample size regarding the participants’ gender, we performed a log-linear analysis with gender and solicitation as independent variables, but gender did not show a significant main effect (χ^2^(1) = 0.14, n.s.) and a significant interaction effect of these (χ^2^(1) = 0.88, n.s.): there was only a significant main effect of solicitation (χ^2^(1) = 8.56, *p* < 0.01).

^3^The mean differences between these two groups of people were found in motivation for restoration of well-being (*t* (173) = 2.76, *p* = 0.006), whereas no significant difference was found in motivation for self-esteem (*t* (173) = 2.20, *p* = 0.029) and motivation for closeness (*t* (173) = 0.15, *p* = 0.877). Note that the t-tests here are set at the significance level of 0.0125, which is equal to 0.05/4.

^4^For more detailed descriptive statistics, including other scales, see the Supplementary Table S1. Note that a conservative alpha level of 0.0125 (which is equal to 0.05/4) was adopted here.

^5^Due to the distributional skewness of our data, the same analysis presented in footnote 2 could only be performed under the unsolicited condition, but as in Study 1, gender did not show a significant main effect (χ^2^(1) = 0.49, n.s.) and interaction effects of these (χ^2^(1) = 0.09, n.s.), there was only a significant main effect of culture (χ^2^(1) = 7.42, *p* < 0.001). Therefore, this paper does not address gender effects.

^6^Considering the effect of repetition, a conservative alpha level of 0.0166 (which is lower than 0.05/3) was adopted here.
