# Supplementary material for: Solicitation matters: Cultural differences in solicited and unsolicited support provision
Source: Front Psychol. 2022 Oct 19;13:953260. doi: 10.3389/fpsyg.2022.953260 (PMC9627144; doi:10.3389/fpsyg.2022.953260)
Supplement: Supplementary file 1 [file Table_1.pdf]

Table S1. The reliability coefficients, means, and standard deviations of the four scales and items by groups in Study 1.

	Support providers (n=113)				Support non-providers (n=62)				Result of Unbalanced ANOVA
	those who were explicitly asked for social support (n=41)		those who were not explicitly asked for social support (n=72)		those who were explicitly asked for social support (n=7)		those who were not explicitly asked for social support (n=55)		
	Mean	*SD*	*Mean*	*SD*	*Mean*	*SD*	*Mean*	*SD*	
1. *Motivation for closeness* (α = .80)	3.44	1.60	3.29	1.39	3.86	1.09	3.24	1.48	F (3, 171) = 0.47, p =.700, η^2 =.01
2. *Motivation for self-esteem* (α = .71)	3.79	1.26	3.66	1.33	3.52	0.92	3.23	1.28	F (3, 171) = 1.79, p =.152, η^2 =.03
3. *Motivation for restoration of well-being* (α = .88)	5.27	1.13	5.10	1.21	5.04	0.88	4.57	1.36	F (3, 171) = 3.00, p =.032, η^2 =.05
4. *Hesitation*	3.22	1.80	3.29	1.52	3.86	1.68	4.22	1.69	F (3, 171) = 4.20, p =.007, η^2 =.07

Note. We did not include data from eight participants in this Supplimental Table (Table S1) because they did not answer the question on whether they provided support. We adopted a conservative alpha level of .0125, which is equal to .05/4.
